# Whole Exome Sequencing Identifies Recessive PKHD1 Mutations in a Chinese Twin Family with Caroli Disease

**DOI:** 10.1371/journal.pone.0092661

**Published:** 2014-04-07

**Authors:** Xiwei Hao, Shiguo Liu, Qian Dong, Hong Zhang, Jing Zhao, Lin Su

**Affiliations:** 1 Pediatric Surgery, The Affiliated Hospital of Medical College, Qingdao University, Qingdao, China; 2 Genetic Laboratory, The Affiliated Hospital of Medical College, Qingdao University, Qingdao, China; Institut de Recerca de la Santa Creu i Sant Pau, Spain

## Abstract

**Background:**

Mutations in *PKHD1* cause autosomal recessive Caroli disease, which is a rare congenital disorder involving cystic dilatation of the intrahepatic bile ducts. However, the mutational spectrum of *PKHD1* and the phenotype-genotype correlations have not yet been fully established.

**Methods:**

Whole exome sequencing (WES) was performed on one twin sample with Caroli disease from a Chinese family from Shandong province. Routine Sanger sequencing was used to validate the WES and to carry out segregation studies. We also described the *PKHD1* mutation associated with the genotype-phenotype of this twin.

**Results:**

A combination of WES and Sanger sequencing revealed the genetic defect to be a novel compound heterozygous genotype in *PKHD1*, including the missense mutation c.2507 T>C, predicted to cause a valine to alanine substitution at codon 836 (c.2507T>C, p.Val836Ala), and the nonsense mutation c.2341C>T, which is predicted to result in an arginine to stop codon at codon 781 (c.2341C>T, p.Arg781*). This compound heterozygous genotype co-segregates with the Caroli disease-affected pedigree members, but is absent in 200 normal chromosomes.

**Conclusions:**

Our findings indicate exome sequencing can be useful in the diagnosis of Caroli disease patients and associate a compound heterozygous genotype in *PKHD1* with Caroli disease, which further increases our understanding of the mutation spectrum of *PKHD1* in association with Caroli disease.

## Introduction

Caroli disease is a rare and complex autosomal congenital disorder that presents as cystic dilatation of the intrahepatic bile ducts [1]. It most commonly manifests as jaundice, cirrhosis, and dilatation of renal tubules, as well as renal impairment in children with associated multicystic or polycystic kidney disease in the second to third decades of life [2], [3]. It can also lead to persistent recurrent cholangitis caused by cholestasis, and, if left untreated, patients will eventually develop biliary cirrhosis, portal hypertension [4], and sometimes bile duct carcinoma [5]. The main mode of Caroli disease inheritance is an autosomal recessive form [6]. Since the causative mutation for autosomal recessive polycystic kidney disease (ARPKD), a major cause of renal and liver-related morbidity and mortality in neonates and infants [7], was identified, several cases of ARPKD with liver manifestations, including Caroli disease, have been reported. Mutations in polycystic kidney and hepatic disease gene 1 (*PKHD1*) are responsible for Caroli disease [8], and many causative mutations are known [9], [10], [11].

*PKHD1* is located on chromosome 6p12.3- 6p12.2 and contains a 16.2 kb coding sequence divided into 66 exons, separated by introns varying in size up to 472 kb [12], [13]. It encodes fibrocystin/polyductin (FPC), a type of membrane-associated receptor-like protein [14], [15] that is predominantly expressed in the apical domain of renal tubule epithelial cells, and may play an important role in collecting duct and biliary differentiation [16]. Recently, advances in next generation sequencing technologies have enabled whole exome sequencing (WES) to become a technically feasible and powerful tool for identifying pathogenic mutations in various Mendelian disorders [17], including rare diseases [18], [19]. This is especially adaptable for the detection of *PKHD1* (including 66 exon) mutations in families with Caroli disease.

To date, the function of PKHD1 is still unclear, and we know little about the relationship between the *PKHD1* genotype and clinical phenotype in Caroli disease. In the present study, therefore, we investigated a Chinese twin family with Caroli disease to detect *PKHD1* mutations using WES, and to evaluate the clinical phenotype correlation associated with these mutations.

## Materials and Methods

### Subjects

The subjects were from a dizygotic male twin family in Shandong province, China. The proband (Patient A, born as the second twin), a 10-year-old boy, the first twin (Patient B), and their parents underwent detailed clinical and ultrasonographical examinations. Both twins were clinically diagnosed with Caroli disease.

The study protocol was approved by the Human Ethics Committee of the Affiliated Hospital of Medical College, Qingdao University (Shandong, China) and is compliant with the Code of Ethics of the World Medical Association and informed consent was obtained. The parents of the subjects in this manuscript have given written informed consent (as outlined in PLOS consent form) to publish these case details. Blood samples were collected from the twins and their parents. DNA was extracted with a standard phenol-chloroform extraction procedure, consisting of the lysis of white blood cells, followed by protein digestion, extraction of the DNA with phenol-chloroform, and precipitation of DNA with isopropanol.

### Whole exome sequencing

WES was carried out on the proband using human exome capture, which was performed according to the protocol from Illumina's TruSeq Exome Enrichment Guide (SureSelectXT Target Enrichment System for Illumina Paired-End Sequencing Library, Agilent). The Agilent Human All Exon 50 Mb Exome Enrichment kit was used as exome enrichment probe sets. Genomic DNA libraries were prepared according to the manufacturer's instructions (Illumina, San Diego, CA). Briefly, 5 µg of genomic DNA in 80 µl of EB buffer was fragmented in a Bioruptor (Diagenode) to 100–500 bp fragments. DNA fragments between 150–250 bp were recovered by gel extraction, then end repair and size selection procedure were performed by T4 DNA poly and Klenow poly cleave 3′. An ‘A’ base was added to the 3′ end using Klenow 3′ to 5′ exo minus, then DNA fragments were ligated to the Illumina multi-PE-adaptor. PCR amplification using 12 cycles was subsequently carried out of the DNA product by mixing it with 1 µl of Illumina multi-PE primer #1 (25 µM), 1 µl of Illumina multi-PE primer #2 (0.5 µM), and 1 µl of Illumina index primer (25 µM).

Captured DNA libraries were sequenced with Illumina HiSeq 2000, which yielded 200 (2×100) bp from the final library fragments using V2 reagent. Base calling was performed by 1.8 software (Illumina; data after 22^nd^ June, 2011). The sequence reads obtained were aligned to the human genome reference sequence (NCBI36/hg18), and variations were identified using the software tool supplied with the instrument. Finally we got 62.09 M high quality reads, and 44.85 M were mapped to the reference genome, the mean depth of the target region was 114.83×. Targeted bases with at least 50× was 75.81%, 20×82.23%, 10×89.04%, 4×93.56%, 1×96.09%. Based on these general statistics, we performed further analysis. All identified *PKHD1* variations were annotated with information to identify candidate mutations displaying the depth of coverage, conservation across species, percentage of reads with the variant, novelty, potential splice site alteration, and likelihood that a variation is deleterious to the protein. This information was extracted from reference data sets or computed in bulk for all variations.

### Mutation analysis and confirmation

Variants of *PKHD1* identified by exome sequencing were confirmed using Sanger sequencing. Two fragments covering the coding sequence and the flanking intronic sequence of *PKHD1* (MIM# 606702, GenBank NM_138694.3) were amplified using *PKHD1* primer pairs for exon 23 (Forward: 5′-CTCCCTTACTGAGTTTCC-3′ and Reverse: 5′-AACAATAAGTCCCTTTCC-3′) and exon 24 (Forward: 5′-GATGAAACTCTGTAAGGTGGAT-3′ and Reverse: 5′-GGAAGGGAGATGTTGGGT-3′). Identical amplification conditions were used for both primer pairs in a total volume of 25 µl containing 250 nM dNTPs, 100 ng of template DNA, 0.5 µM of each primer, and 1.25 U AmpliTaq Gold DNA polymerase in 1× reaction buffer (10 mM Tris HCl, pH 8.3, 50 mM KCl, 2.5 mM MgCl_2_). PCR amplifications were performed with an initial denaturing step at 94°C for 5 min, then 35 cycles of: 94°C for 30 s, 59°C or 63°C (for exons 23 and 24, respectively) for 60 s, 72°C for 30 s, followed by 10 min of final extension at 72°C. Amplified PCR products were purified and sequenced using the appropriate PCR primers and the BigDye Terminator Cycle Sequencing kit (Applied Biosystems, Foster City, CA) and run on an automated sequencer, ABI 3730XL (Applied Biosystems) to perform mutational analysis.

### Denaturing high-performance liquid chromatography (DHPLC) screening of the *PKHD1* mutation

Mutation screening of the fragments harboring the c.2341 C>T and c.2507 T>C mutations in exons 23 and 24 of *PKHD1* was performed with denaturing high-performance liquid chromatography (DHPLC Wave DHPLC; Transgenomic, Omaha, NE) in 100 normal controls. DHPLC used an initial concentration of 48% buffer A (0.1 M triethylammonium acetate (TEAA; Transgenomic) and 52% buffer B (0.1 M TEAA containing 25% acetonitrile; Transgenomic) at 65°C. Data were analyzed by comparing the chromatograms.

## Results

### Clinical phenotype

The proband (Patient A) was healthy until the age of 1 year, when he was found to have asymptomatic splenomegaly. At 5 years of age he presented with anorexia and an upper abdominal mass and was diagnosed with Caroli disease. After 2 years, mild jaundice was apparent on the systemic skin, and the abdominal mass had increased, causing upper abdominal pain. This was accompanied by liver cirrhosis, hypersplenism, severe anemia, and a polycystic kidney. The first twin (Patient B) had no obvious clinical symptoms except for intrahepatic bile duct dilatation. Both parents were negative for the presence of liver and renal anomalies as shown by an ultrasonography, were non-consanguineous, and had no family history of genetic diseases.

### Mutation analysis

**Figure 1:**
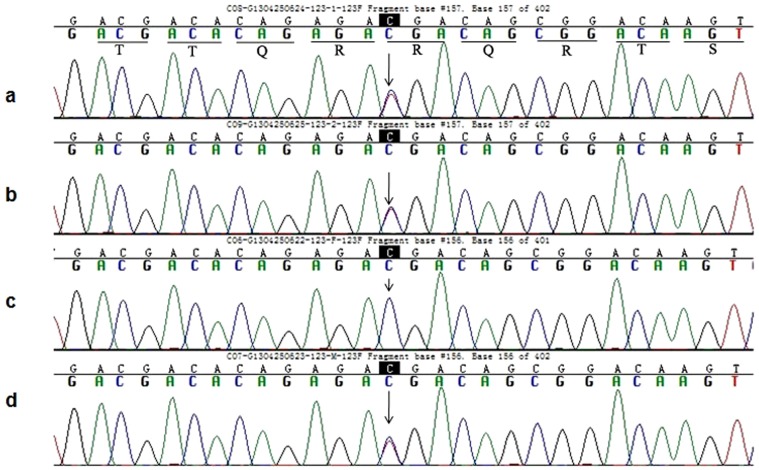
Partial sequence of exon 23 in the *PKHD1* from member of this Caroli disease-affected pedigree. (a), (b) and (d) Arrowhead indicates the heterozygous C and T at nucleotide 2341 in proband, elder twins and their mother respectively. (c) Arrowhead indicates the C at nucleotide 2341(wild type) in their father.

**Figure 2:**
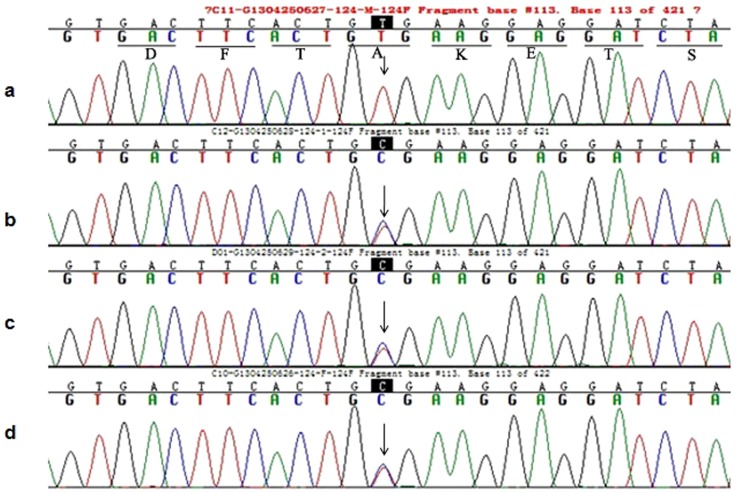
Partial sequence of exon 24 in the *PKHD1* from member of this Caroli disease-affected pedigree. (b), (c) and (d) Arrowhead indicates the heterozygous T and C at nucleotide 2507 in proband, elder twins and their father respectively. (a) Arrowhead indicates the T at nucleotide 2507 (wild type) in their mother.

As *PKHD1* is a long gene, composed of 66 exons, we performed WES and applied several filtering steps to exclude nongenetic variants by filtering the database of dbSNP and 1000 genomes to select for nucleotide changes predicted to have a damaging effect on the PKHD1 protein by SIFT (Sorting intolerant from tolerant) and PolyPhen-2. The depth of coverage for c.2341 C>T and c.2507 T>C mutations in exons 23 and 24 of *PKHD1* are 77× and 105×, which suggest high reliability of sequencing. Sanger sequencing confirmation of the proband revealed a compound heterozygous genotype, based on a novel missense variant, c.2507 T>C (p.Val836Ala, SIFT score 0.02, PolyPhen-2 score 0.998), predicted to cause a valine to alanine substitution at codon 836 in exon 24 (accession no. NM_138694.3; first nucleotide of the initiation codon numbered 1), and a known nonsense mutation, c.2341C>T (p.Arg781*), predicted to change an arginine to a stop codon at codon 781 in exon 23 (Figures 1, 2). Mutation p.Arg781* has previously been described in Caucasian-American patients and those from the Netherlands, France, Denmark, Germany, Portugal, and Belgium.

Co-segregation analysis of this pedigree revealed that the first twin also carries the same compound heterozygous genotype, and that both parents are carriers of a single heterozygous mutation. The father harbors the p.Val836Ala mutant, while the mother carries the p.Arg781* variant. These two different missense variants were individually inherited from both parents, resulting in the compound heterozygous genotype co-segregating with the Caroli disease-affected pedigree in both twins.

Comparing the WES findings with the different phenotypes of the twins, we determined whether any variants in genes related to Caroli disease, hepatic function, or kidney function could act as a genetic modifier for Caroli disease (such as *NPHP3*, *PKD1* and so on). However, no positive results were identified.

### DHPLC screening of the *PKHD1* mutation

**Figure 3:**
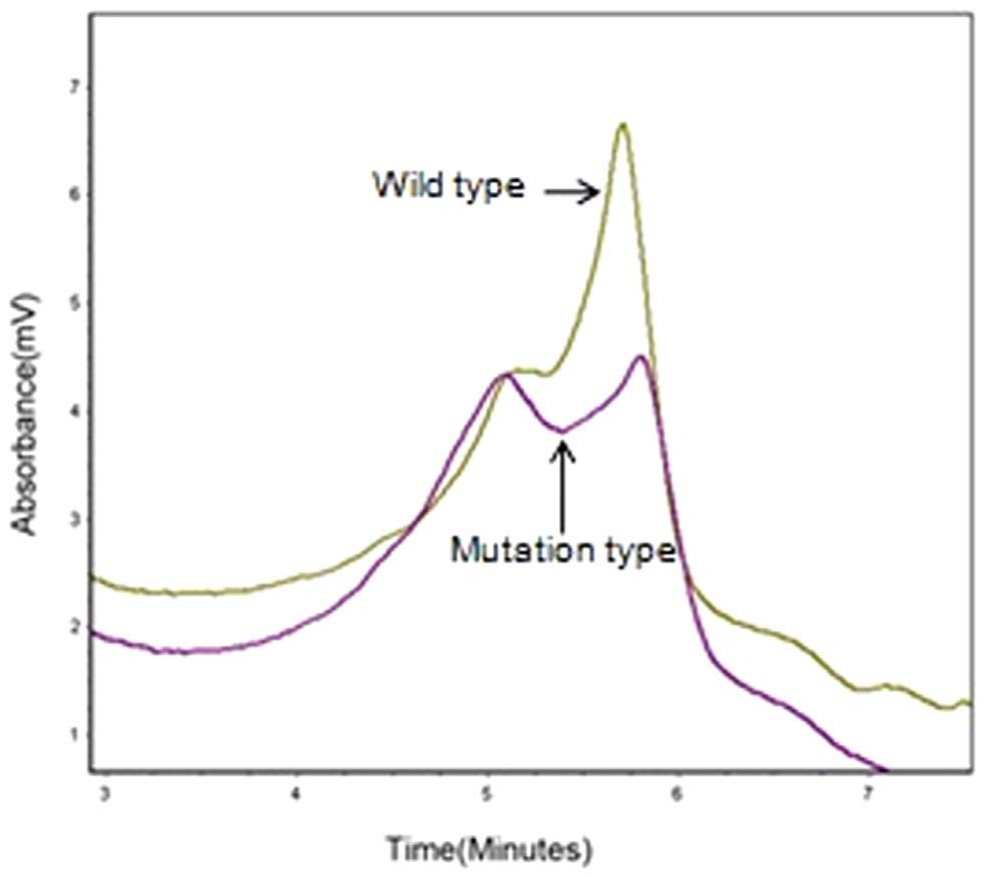
DHPLC shows wave pattern of wild type and mutant type *PKHD1*. Wild type: from normal individuals. Mutant type : from affected individuals.

Analysis of 200 normal chromosomes from 100 healthy controls of Chinese Han origin by DHPLC found no evidence of the novel missense variant, c.2507T>C (p.Val836Ala), or the p.Arg781* variant (Figure 3). This suggests that the compound heterozygous genotype observed in this family is causative of the Caroli disease phenotype.

### Bioinformatic analysis of *PKHD1* mutation

**Figure 4:**
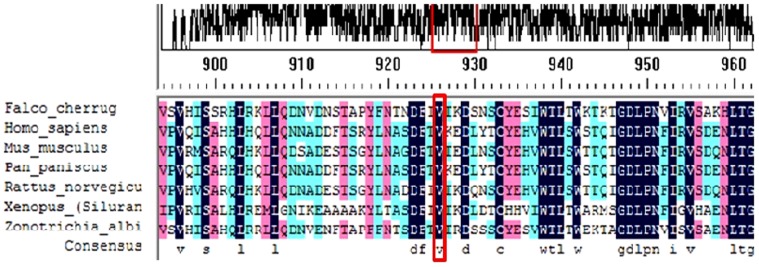
Multiple-sequence alignment of the PKHD1 protein including *Mus musculus*, *Rattus norvegicus*, *Pan paniscus*, *Xenopus (Silurana) tropicalis*, *Falco cherrug*, *Zonotrichia albicollis* and *Homo sapiens*. The Val 836 residue is located within a highly conserved region.

We obtained PKHD1 family protein sequences from NCBI and UCSC websites and used Vector NTI software to perform multiple-sequence alignments in various animal species, including *Mus musculus*, *Rattus norvegicus*, *Pan paniscus*, *Xenopus (Silurana) tropicalis*, *Falco cherrug*, *Zonotrichia albicollis* and *Homo sapiens* (Figure 4). The p.Val836Ala variant was found to be located in a highly conserved region of the PKHD1 protein.

## Discussion

Caroli disease, which has an estimated incidence of approximately 1 in 100,000 newborns, is a complex disorder of the intrahepatic bile ducts presenting with multiple saccular segmental and cystic dilatations [20]. When progressive, it can cause recurrent cholangitis, jaundice, the accumulation of intrahepatic stones, portal hypertension, liver failure, and even cholangiocarcinoma [4], [21]. The pathogenesis of Caroli disease appears to be related to dilatations and malformation of a ductal plate, which are either diffuse or confined to only one part of the liver [22]. It can be divided into the pure form of Caroli disease and Caroli's syndrome, which presents with repeated bouts of cholangitis resulting from bile stasis, hepatolithiasis, gall bladder stones, and symptoms associated with hepatic fibrosis such as portal hypertension and poor hepatic reserve. The disease spectrum of clinical phenotypes caused by mutations in *PKHD1* is relatively complex, ranging from perinatally-fatal ARPKD to Congenital Hepatic Fibrosis (CHF)-predominant presentations in adulthood with mild or no apparent kidney disease [23].

In 2002, Ward and colleagues first screened *PKHD1* mutations in 14 probands with ARPKD (some cases of ARPKD with mainly liver manifestations, including Caroli disease diagnosed in adulthood), and revealed that eight of the affected individuals were compound heterozygotes [16]. Since then, several large-scale mutation detection studies have focused on the longest *PKHD1* ORF (open reading frame) [24], [25], [26]. Mera et al. used direct sequencing to detect *PKHD1* mutations in a cohort of 90 North American ARPKD/CHF patients, and identified 77 *PKHD1* sequence variants, which supported previously published genotype-phenotype correlation findings [23]. Sandro et al. used DHPLC to report a compound heterozygous genotype (c.10364delC/p.Ile 3468Val) of *PKHD1* in a 36-year-old female with Caroli disease [27]. To date, at least 300 different pathogenic mutations have been found throughout most of the coding exons of the human *PKHD1* gene (http://www.humgen.rwth-aachen.de/). Approximately 60% of these are truncating mutations and 40% are missense mutations, suggesting that the recessive form of this disease results from loss of function of the normal protein in different degree [28].

Although severely affected ARPKD and CHF patients account for most known *PKHD1* mutations, and patients with Caroli disease have a low rate of *PKHD1* mutation detection [27], the genotype-phenotype associated with *PKHD1* mutations is relatively complex. ARPKD patients carrying two truncating mutations have a severe disease phenotype resulting in perinatal death, while other combinations of mutations, such as splicing and missense mutations, have a more variable but usually less severe phenotype [29]. Meral et al. analyzed the clinical, molecular, *PKHD1* mutation, and imaging data of 73 patients with ARPKD and CHF (including 51 with Caroli syndrome). Although biallelic *PKHD1* mutations were identified in only 43 families and one heterozygous mutation in 20 families, the authors concluded that kidney and liver disease are independent, and that variability in severity does not reflect the type of *PKHD1* mutation [11].

In this work, we identified a causative compound heterozygous *PKHD1* mutation in a Chinese twin family with Caroli disease. Because of the large size of *PKHD1*, DHPLC or single-strand conformation polymorphism (SSCP) screening techniques have been used in all but one of the published studies; variants detected by screening were further characterized by targeted direct sequencing. However, DHPLC and SSCP have their own shortcomings, so, more recently, next generation sequencing technologies have been employed to rapidly accelerate the discovery of the genetic causes of human diseases. WES is a powerful tool for investigating the genetic underpinnings of human disease [30]. As Mendelian pathogenic mutations are frequently exonic, exome sequencing is an efficient method to simultaneously examine many coding regions, and has rapidly proven to be an important tool in genetic research [31]. It is especially adapted to large genes such as *PKHD1*, as it not only overcomes the time-consuming and laborious nature of traditional PCR but also has relatively lower costs. Therefore, we performed WES to screen *PKHD1* mutations.

We found a novel compound heterozygous genotype of *PKHD1* (p.Val836Ala/p.Arg781*) in twin of a Chinese family with Caroli disease. Their father harbors the p.Val836Ala mutant, while their mother carries the p.Arg781* variant. The p.Val836Ala mutation results in an amino acid change from valine to alanine, but as both amino acids are neutral the potential impairment on protein function is unclear; however, the amino acid at position 836 is highly conserved between species. The p.Arg781* variation leads to a truncated PKHD1 protein, which lacks the succeeding seven IPT domains and two G8 domains, thus losing the functionality of the wild-type protein. Zerres et al. firstly reported p.Arg781* in ARPKD [7], showing that the same mutation can cause different clinical phenotypes.

As seen in the present family, the proband's clinical phenotype of Caroli syndrome included liver cirrhosis, hypersplenism, severe anemia, and a polycystic kidney, while the first twin suffered from pure Caroli disease. Therefore, the genotype-phenotype associated with *PKHD1* mutations is complex. Sgro et al. [9] reported the compound heterozygous genotype *PKHD1* IVS55+1G→A/p.Trp 2690Arg in patients detected prenatally with Caroli disease; IVS55+1G→A was inherited from the father and the missense mutation p.Trp2690Arg from the mother, which is consistent with a recessive pattern of inheritance. Our findings also lay the basis for a more accurate and rapid prenatal diagnosis of Caroli disease in its early stages.

In conclusion, we combined WES with Sanger sequencing to report a novel compound heterozygous genotype of *PKHD1* causative of Caroli disease in a Chinese twin family. The characteristic disease phenotype shows obvious differences between the twins. Our study enlarged the genotype-phenotype correlations of *PKHD1*, which might be useful for understanding the pathophysiological mechanisms of Caroli disease.

## References

[1] Desmet VJ (1992) What is congenital hepatic fibrosis. Histopathology 20: 465–477. 1607148 10.1111/j.1365-2559.1992.tb01031.x

[2] Desmet VJ (1998) Ludwig symposium on biliary disorders–part I. Pathogenesis of ductal plate abnormalities. Mayo Clinic proceedings. Mayo Clinic 73: 80–89. 10.4065/73.1.80 9443684

[3] Keane F, Hadzic N, Wilkinson ML, Qureshi S, Reid C, et al (1997) Neonatal presentation of Caroli's disease. Arch Dis Child Fetal Neonatal Ed 77: F145–146. 9377141 10.1136/fn.77.2.f145 PMC1720696

[4] Taylor AC, Palmer KR (1998) Caroli's disease. Eur J Gastroenterol Hepatol 10: 105–108. 9581983 10.1097/00042737-199802000-00001

[5] Falco E, Nardini A, Celoria G, Briglia R, Stefani R, et al (1993) [Caroli's disease associated with cholangiocarcinoma. A case of our own observation]. Minerva chirurgica 48: 961–964. 8290138

[6] Mousson C, Rabec M, Cercueil JP, Virot JS, Hillon P, et al (1997) Caroli's disease and autosomal dominant polycystic kidney disease: a rare association. Nephrology, dialysis, transplantation : official publication of the European Dialysis and Transplant Association - European Renal Association 12: 1481–1483. 10.1093/ndt/12.7.1481 9249792

[7] Zerres K, Mucher G, Becker J, Steinkamm C, Rudnik-Schoneborn S, et al (1998) Prenatal diagnosis of autosomal recessive polycystic kidney disease (ARPKD): molecular genetics, clinical experience, and fetal morphology. American journal of medical genetics 76: 137–144. 9511976

[8] Ward CJ, Hogan MC, Rossetti S, Walker D, Sneddon T, et al (2002) The gene mutated in autosomal recessive polycystic kidney disease encodes a large, receptor-like protein. Nat Genet 30: 259–269. 11919560 10.1038/ng833

[9] Sgro M, Rossetti S, Barozzino T, Toi A, Langer J, et al (2004) Caroli's disease: prenatal diagnosis, postnatal outcome and genetic analysis. Ultrasound in obstetrics & gynecology : the official journal of the International Society of Ultrasound in Obstetrics and Gynecology 23: 73–76. 10.1002/uog.943 14971004

[10] Rossetti S, Torra R, Coto E, Consugar M, Kubly V, et al (2003) A complete mutation screen of PKHD1 in autosomal-recessive polycystic kidney disease (ARPKD) pedigrees. Kidney Int 64: 391–403. 12846734 10.1046/j.1523-1755.2003.00111.x

[11] Gunay-Aygun M, Font-Montgomery E, Lukose L, Tuchman GM, Piwnica-Worms K, et al (2013) Characteristics of congenital hepatic fibrosis in a large cohort of patients with autosomal recessive polycystic kidney disease. Gastroenterology 144: 112–121.e2. 23041322 10.1053/j.gastro.2012.09.056 PMC4162098

[12] Lens XM, Onuchic LF, Wu G, Hayashi T, Daoust M, et al (1997) An integrated genetic and physical map of the autosomal recessive polycystic kidney disease region. Genomics 41: 463–466. 9169147 10.1006/geno.1997.4671

[13] Zerres K, Mucher G, Bachner L, Deschennes G, Eggermann T, et al (1994) Mapping of the gene for autosomal recessive polycystic kidney disease (ARPKD) to chromosome 6p21-cen. Nat Genet 7: 429–432. 7920664 10.1038/ng0794-429

[14] Gunay-Aygun M, Avner ED, Bacallao RL, Choyke PL, Flynn JT, et al (2006) Autosomal recessive polycystic kidney disease and congenital hepatic fibrosis: summary statement of a first National Institutes of Health/Office of Rare Diseases conference. J Pediatr 149: 159–164. 16887426 10.1016/j.jpeds.2006.03.014 PMC2918414

[15] Wang S, Zhang J, Nauli SM, Li X, Starremans PG, et al (2007) Fibrocystin/polyductin, found in the same protein complex with polycystin-2, regulates calcium responses in kidney epithelia. Mol Cell Biol 27: 3241–3252. 17283055 10.1128/MCB.00072-07 PMC1899915

[16] Ward CJ, Hogan MC, Rossetti S, Walker D, Sneddon T, et al (2002) The gene mutated in autosomal recessive polycystic kidney disease encodes a large, receptor-like protein. Nat Genet 30: 259–269. 11919560 10.1038/ng833

[17] Ng SB, Buckingham KJ, Lee C, Bigham AW, Tabor HK, et al (2010) Exome sequencing identifies the cause of a mendelian disorder. Nat Genet 42: 30–35. 19915526 10.1038/ng.499 PMC2847889

[18] Foo JN, Liu JJ, Tan EK (2012) Whole-genome and whole-exome sequencing in neurological diseases. Nat Rev Neurol 8: 508–517. 22847385 10.1038/nrneurol.2012.148

[19] Riviere JB, van Bon BW, Hoischen A, Kholmanskikh SS, O'Roak BJ, et al (2012) De novo mutations in the actin genes ACTB and ACTG1 cause Baraitser-Winter syndrome. Nat Genet 44: 440–444, S1–2. 22366783 10.1038/ng.1091 PMC3677859

[20] Kassahun WT, Kahn T, Wittekind C, Mossner J, Caca K, et al (2005) Caroli's disease: liver resection and liver transplantation. Experience in 33 patients. Surgery 138: 888–898. 16291390 10.1016/j.surg.2005.05.002

[21] Totkas S, Hohenberger P (2000) Cholangiocellular carcinoma associated with segmental Caroli's disease. European journal of surgical oncology : the journal of the European Society of Surgical Oncology and the British Association of Surgical Oncology 26: 520–521. 10.1053/ejso.1999.0936 11016478

[22] Desmet VJ (1998) Ludwig symposium on biliary disorders–part I. Pathogenesis of ductal plate abnormalities. Mayo Clinic proceedings. Mayo Clinic 73: 80–89. 10.4065/73.1.80 9443684

[23] Gunay-Aygun M, Tuchman M, Font-Montgomery E, Lukose L, Edwards H, et al (2010) PKHD1 sequence variations in 78 children and adults with autosomal recessive polycystic kidney disease and congenital hepatic fibrosis. Mol Genet Metab 99: 160–173. 19914852 10.1016/j.ymgme.2009.10.010 PMC2818513

[24] Bergmann C, Senderek J, Windelen E, Kupper F, Middeldorf I, et al (2005) Clinical consequences of PKHD1 mutations in 164 patients with autosomal-recessive polycystic kidney disease (ARPKD). Kidney Int 67: 829–848. 15698423 10.1111/j.1523-1755.2005.00148.x

[25] Bergmann C, Senderek J, Sedlacek B, Pegiazoglou I, Puglia P, et al (2003) Spectrum of mutations in the gene for autosomal recessive polycystic kidney disease (ARPKD/PKHD1). J Am Soc Nephrol 14: 76–89. 12506140 10.1097/01.asn.0000039578.55705.6e

[26] Sharp AM, Messiaen LM, Page G, Antignac C, Gubler MC, et al (2005) Comprehensive genomic analysis of PKHD1 mutations in ARPKD cohorts. J Med Genet 42: 336–349. 15805161 10.1136/jmg.2004.024489 PMC1736033

[27] Rossetti S, Torra R, Coto E, Consugar M, Kubly V, et al (2003) A complete mutation screen of PKHD1 in autosomal-recessive polycystic kidney disease (ARPKD) pedigrees. Kidney Int 64: 391–403. 12846734 10.1046/j.1523-1755.2003.00111.x

[28] Bergmann C, Kupper F, Dornia C, Schneider F, Senderek J, et al (2005) Algorithm for efficient PKHD1 mutation screening in autosomal recessive polycystic kidney disease (ARPKD). Hum Mutat 25: 225–231. 15706593 10.1002/humu.20145

[29] Denamur E, Delezoide AL, Alberti C, Bourillon A, Gubler MC, et al (2010) Genotype-phenotype correlations in fetuses and neonates with autosomal recessive polycystic kidney disease. Kidney Int 77: 350–358. 19940839 10.1038/ki.2009.440

[30] Ng SB, Buckingham KJ, Lee C, Bigham AW, Tabor HK, et al (2010) Exome sequencing identifies the cause of a mendelian disorder. Nat Genet 42: 30–35. 19915526 10.1038/ng.499 PMC2847889

[31] Biesecker LG, Shianna KV, Mullikin JC (2011) Exome sequencing: the expert view. Genome Biol 12: 128. 21920051 10.1186/gb-2011-12-9-128 PMC3308041

